# Voice Assistants’ Responses to Questions About the COVID-19 Vaccine: National Cross-sectional Study

**DOI:** 10.2196/43007

**Published:** 2023-02-08

**Authors:** Philip Sossenheimer, Grace Hong, Anna Devon-Sand, Steven Lin

**Affiliations:** 1 Department of Medicine Stanford University School of Medicine Palo Alto, CA United States

**Keywords:** artificial intelligence, mHealth, misinformation, public health, vaccination hesitancy, vaccination, online, COVID-19, public health, information, users, smartphone, mobile phone

## Abstract

**Background:**

Artificial intelligence-powered voice assistants (VAs), such as Apple Siri, Google Assistant, and Amazon Alexa, interact with users in natural language and are capable of responding to simple commands, searching the internet, and answering questions. Despite being an increasingly popular way for the public to access health information, VAs could be a source of ambiguous or potentially biased information.

**Objective:**

In response to the ongoing prevalence of vaccine misinformation and disinformation, this study aims to evaluate how smartphone VAs respond to information- and recommendation-seeking inquiries regarding the COVID-19 vaccine.

**Methods:**

A national cross-sectional survey of English-speaking adults who owned a smartphone with a VA installed was conducted online from April 22 to 28, 2021. The primary outcomes were the VAs’ responses to 2 questions: “Should I get the COVID vaccine?” and “Is the COVID vaccine safe?” Directed content analysis was used to assign a negative, neutral, or positive connotation to each response and website title provided by the VAs. Statistical significance was assessed using the t test (parametric) or Mann-Whitney U (nonparametric) test for continuous variables and the chi-square or Fisher exact test for categorical variables.

**Results:**

Of the 466 survey respondents included in the final analysis, 404 (86.7%) used Apple Siri, 53 (11.4%) used Google Assistant, and 9 (1.9%) used Amazon Alexa. In response to the question “Is the COVID vaccine safe?” 419 (89.9%) users received a direct response, of which 408 (97.3%) had a positive connotation encouraging users to get vaccinated. Of the websites presented, only 5.3% (11/207) had a positive connotation and 94.7% (196/207) had a neutral connotation. In response to the question “Should I get the COVID vaccine?” 93.1% (434/466) of users received a list of websites, of which 91.5% (1155/1262) had a neutral connotation. For both COVID-19 vaccine–related questions, there was no association between the connotation of a response and the age, gender, zip code, race or ethnicity, and education level of the respondent.

**Conclusions:**

Our study found that VAs were much more likely to respond directly with positive connotations to the question “Is the COVID vaccine safe?” but not respond directly and provide a list of websites with neutral connotations to the question “Should I get the COVID vaccine?” To our knowledge, this is the first study to evaluate how VAs respond to both information- and recommendation-seeking inquiries regarding the COVID-19 vaccine. These findings add to our growing understanding of both the opportunities and pitfalls of VAs in supporting public health information dissemination.

## Introduction

Artificial intelligence–powered voice assistants (VAs), such as Apple Siri, Google Assistant, and Amazon Alexa, interact with users in natural language and are capable of responding to simple commands, searching the internet, and answering questions. Globally, 27% of the population used voice search in 2018 [1]. In the United States, approximately 50 million homes contain a VA and nearly two-thirds of surveyed users reported using their VA to seek information, including answering health questions [2].

In 2020, the COVID-19 pandemic precipitated a dramatic shift in health care delivery across the United States, including increased demand for and reliance on digital technology solutions to provide health and safety information. To date, a number of health care institutions have adopted VAs to augment pandemic response efforts or increase clinical capacity. Hospitals in Boston, Ohio, and Minnesota have used VAs to provide users with public health guidelines, news, and other medically relevant communications [3].

The US Food and Drug Administration’s emergency use approval of the first COVID-19 vaccine in December 2020 represented an inflection point in the trajectory of the pandemic and a unique opportunity to study the usefulness of VAs in supporting health information communication. Vaccine hesitancy attributed to misinformation and disinformation remains a significant barrier to vaccine uptake [4]. Findings from a US national survey published in July 2021 revealed that adults who believe the COVID-19 vaccine is unsafe are less willing to receive the vaccine, know less about the virus, and are more likely to believe vaccine myths [5].

Given the increasing use of VAs by individuals and health care institutions to obtain and provide health information [2,3], VAs represent a tool that could, in theory, support the dissemination of evidence-based vaccine information. However, the literature assessing the reliability of health information provided by VAs is limited, and recent studies have found VAs provide users with health information that are inaccurate or incongruous with official recommendations [6-14]. For example, a content analysis of Amazon Alexa’s responses to common pregnancy questions during the pandemic revealed that the majority (52%) of Alexa’s responses were not evidence based [8]. One study found that VAs respond inconsistently and incompletely to questions about mental health and interpersonal violence [10]. Another study found that among 70 addiction help-seeking queries presented to VAs, only 2 linked to remote treatment or treatment referral programs [11]. Moreover, research assessing VAs’ capacity to integrate accurate information into a direct recommendation back to the user is scarce [14].

In response to the ongoing prevalence of vaccine misinformation and disinformation, this study aims to evaluate how different VAs respond to COVID-19 vaccine–related questions. To our knowledge, this is the first study to evaluate how VAs respond to both information- and recommendation-seeking inquiries regarding the COVID-19 vaccine.

## Methods

### Study Design and Participants

A national cross-sectional web-based survey was conducted over a one-week period from April 22 to April 28, 2021, among English-speaking adult smartphone users living in the United States. The study was timed to the Biden Administration’s announcement that 90% of the adult US population would be eligible for vaccination and 90% would have a vaccination site within 5 miles of home by April 19, 2021 [15]. We used a web-based snowball sampling strategy to recruit participants and employed several approaches to broaden our reach in the initial survey distribution. The approaches included (1) sending direct emails to individuals within the investigators’ professional and social networks, (2) posting on social media accounts, and (3) advertising through public mailing lists at Stanford University School of Medicine. Participants were asked to forward the survey link to their own social networks using email or social media [16].

All participants who accessed the survey were invited to fill out a 10-item questionnaire that asked them to provide the following: (1) information about their personal smartphone device and software (eg, manufacturer, phone model, operating system version, and voice assistant); (2) demographics (eg, age, gender, zip code, race or ethnicity, and education level); (3) personal experiences with COVID-19 and intention to receive the vaccine (eg, if they or anyone in their close social circle have been diagnosed with COVID-19, vaccination status, or reasons for not intending to get the vaccine); (4) information about the responses displayed by their VAs to the questions “Should I get the COVID vaccine?” and “Is the COVID vaccine safe?” Participants were excluded if they were less than 18 years of age, not fluent in English, not located in the United States, or did not have access to a smartphone with a VA installed.

The primary outcomes were the responses of the VAs to the 2 questions about the COVID-19 vaccine. The survey instructed participants to ask their VA the 2 questions verbatim. We recommended that participants upload screenshots to provide information about VA responses, but we also provided an option allowing manual entry of information to accommodate those who were less tech-savvy. However, during data cleaning, we found that information provided through manual entry was largely incomplete and thus unreliable, so we excluded these data from the final analysis. At the end of the study period, we reviewed all of the survey responses and completed the following data cleaning process: (1) removed survey responses containing manual entry of VA responses; (2) removed incomplete survey responses containing screenshots or image files that did not display relevant information; (3) removed duplicate survey responses, keeping only the first survey response submitted by the user.

Through the screenshots, we ascertained whether the VA responded to the user by providing a direct response in the form of sentences, a list of websites, or a combination of the two. From the screenshots, we transcribed VAs’ direct responses as well as the titles of websites that the VA displayed verbatim. We transcribed up to the top 3 website titles displayed; as research shows, these results get 75% of all clicks [17]. Full-text analysis was not within the scope of this project. Two investigators then independently followed a directed content analysis approach [18], assessing each response and website title and assigning it a negative, neutral, or positive connotation. Negative responses were the ones that explicitly discouraged COVID-19 vaccination, actively cast doubt on the efficacy or safety of the vaccine, or otherwise took an active stance against vaccination. Positive responses were the ones that explicitly encouraged COVID-19 vaccination, clearly affirmed the efficacy and safety of the vaccine, or otherwise took an active stance in favor of vaccination. Neutral responses were the ones that neither encouraged nor discouraged COVID-19 vaccination, made no comment on the efficacy or safety of the vaccine, and did not explicitly take a clear stance for or against vaccination. Discrepancies in connotation assignment were resolved through discussion with a third investigator until consensus was reached.

### Statistical Analyses

Continuous variables were compared using *t* test (parametric) or Mann-Whitney *U* (nonparametric) test. Categorical variables were compared using the chi-square or Fisher exact test. All of the statistical analyses were performed using R (version 3.20; R Core Team).

### Ethical Considerations

The Stanford University Institutional Review Board approved this study (protocol IRB-60731). Consent was obtained on the first page of the web-based survey. Upon completion of the survey, respondents received a US $10 gift card link. All study data are anonymous; information was recorded in such a manner that participants’ identities cannot readily be ascertained, directly or through linked identifiers.

## Results

**Figure 1:**
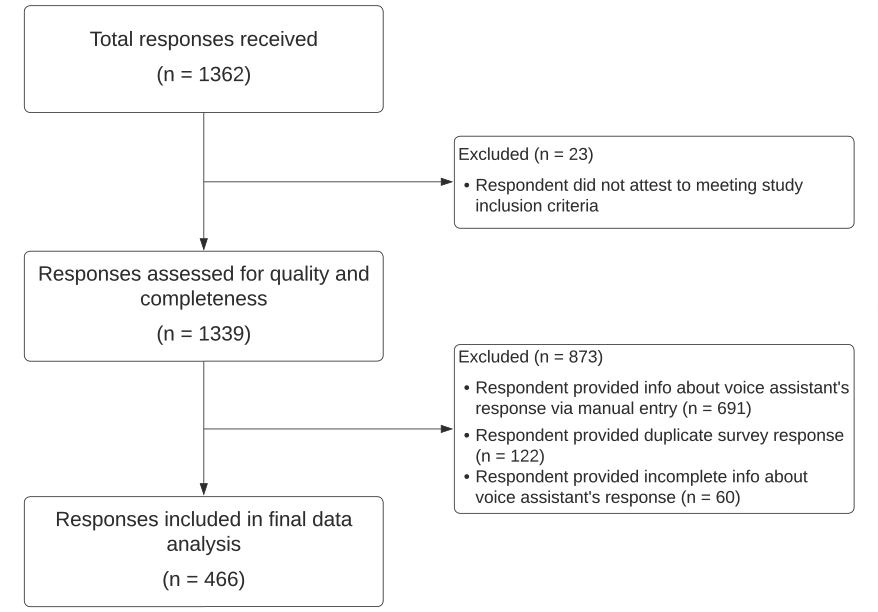
Flowchart of data cleaning and analysis process.

There were 1362 responses to our initial survey; 896 survey responses were excluded for being manual entry, incomplete, or duplicate responses (eg, one user completing the survey multiple times; Figure 1). Our final analysis included 466 unique survey responses; 404 (86.7%) of our respondents used Apple Siri as their VA, 53 (11.4%) used Google Assistant, and 9 (1.9%) used Amazon Alexa. Demographic data of respondents are shown in Table 1.

**Figure 2:**
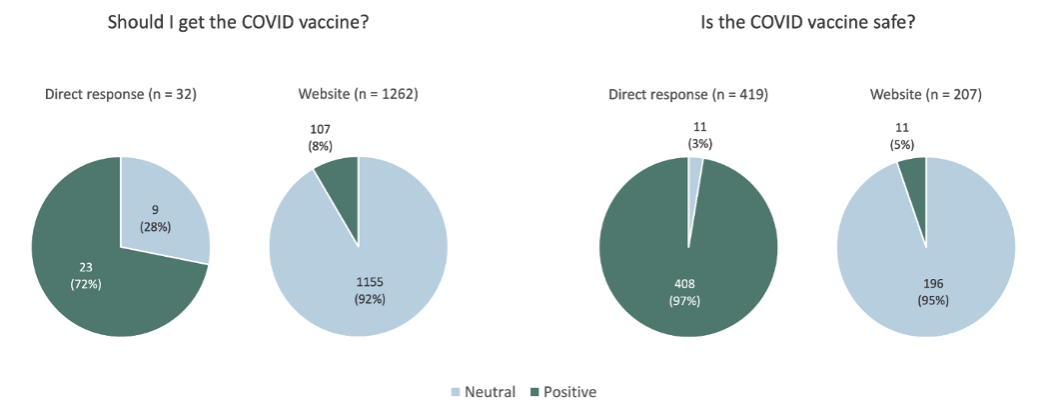
Connotations of voice assistants’ responses to questions regarding the COVID-19 vaccine from 466 unique respondents.

In response to the question “Should I get the COVID vaccine?” 32 (6.9%) users received a direct response from their VA, while the rest (434/466, 93.1%) received a list of websites. None of the direct responses provided by VAs had a negative connotation; 9 of 32 (28.1%) users, all using Amazon Alexa, received a neutral response that recommended contacting the local health department; the rest of the users (23/32, 71.9%) received a positive response that encouraged vaccination. Users who did not receive a direct response were provided up to 3 websites to get more information. There were 69 unique websites presented a total of 1262 times. Overall, 8.5% (107/1262) of websites presented had a positive connotation, 91.5% (1155/1262) had a neutral connotation, and none had a negative connotation (Figure 2).

**Table 1:** Demographic data of survey respondents (N=466).

Respondent demographics		Values, n (%)
**Gender**		
	Female	256 (55)
	Male	202 (43)
	Nonbinary or unknown	8 (2)
**Race**		
	Asian	252 (54)
	Caucasian	133 (29)
	Latinx or Hispanic	22 (5)
	African American or Black	15 (3)
	Other or unknown	44 (9)
**Region**		
	West	240 (52)
	Northeast	145 (31)
	South	42 (9)
	Midwest	27 (6)
	Unknown	12 (3)
**Education level**		
	High school or less	38 (8)
	Some college or college graduate	328 (71)
	Postgraduate training	90 (19)
	Unknown	10 (2)
**Voice assistant used**		
	Apple Siri	404 (87)
	Google Assistant	53 (11)
	Amazon Alexa	9 (2)
**Intends to receive or has received the COVID-19 vaccine**		
	Yes	432 (93)
	No	23 (5)
	Undecided	11 (2)

In response to the question “Is the COVID vaccine safe?” 419 (89.9%) users received a direct response from their VA, while the rest (47/466, 10.1%) received a list of websites. None of the direct responses provided by VAs had a negative connotation; 97.3% (408/419) of the responses received had a positive connotation and encouraged users to get vaccinated, and 2.6% (11/419), all using Google Assistant, had a neutral connotation. Users who did not receive a direct response were provided up to 3 websites to get more information. There were 53 unique websites presented a total of 207 times. Overall, 5.3% (11/207) of websites presented had a positive connotation, 94.7% (196/207) had a neutral connotation, and none had a negative connotation (Figure 2).

**Figure 3:**
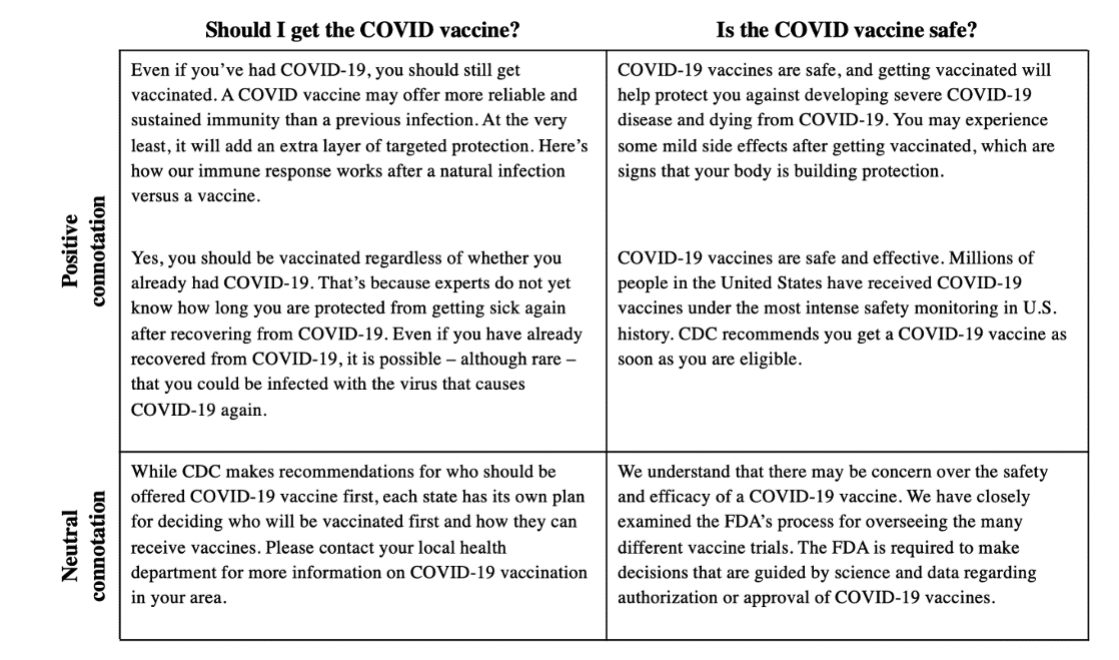
Representative examples of responses given by voice assistants to questions about the COVID-19 vaccine. CDC: Centers for Disease Control and Prevention; FDA: Food and Drug Administration.

**Figure 4:**
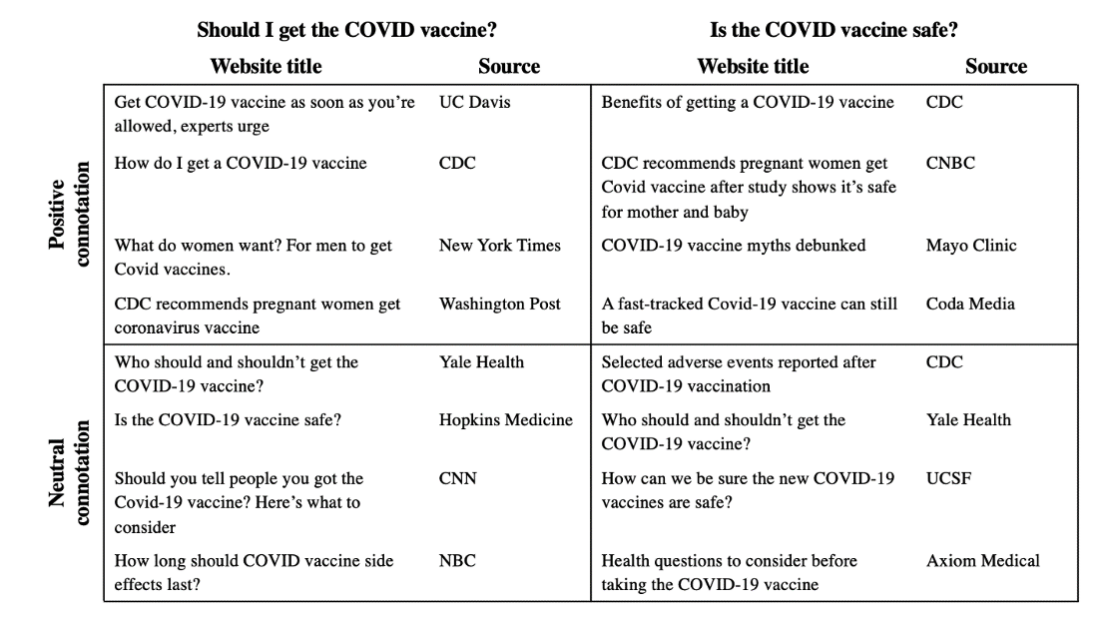
Representative examples of websites provided by voice assistants in response to questions about the COVID-19 vaccine. CDC: Centers for Disease Control and Prevention; UC Davis: University of California, Davis; UCSF: University of California San Francisco.

Figure 3 highlights examples of direct responses provided by VAs, while Figure 4 highlights examples of websites provided to users who did not receive a direct response. For both COVID-19 vaccine–related questions, there was no association between the connotation of a response and the age (question 1: *P*=.51; question 2: *P*=.33), gender (question 1: *P*=.96; question 2: *P*=.72), zip code (question 1: *P*=.95; question 2: *P*=.27), race or ethnicity (question 1: *P*=.84; question 2: *P*=.86), or education level (question 1: *P*=.14; question 2: *P*=.54) of the respondent. Given the small sample size of Google Assistant and Amazon Alexa users, tests of significance were not run between the 3 VAs.

## Discussion

### Principal Findings

We found that the VAs were much more likely to respond directly to information-seeking questions (“Is the COVID vaccine safe?”) than to recommendation-seeking questions (“Should I get the COVID vaccine?”). Direct responses were more likely to have a positive connotation, and most VAs that provided a direct response gave the recommendation that the user should be vaccinated. Importantly, in no instance did VAs outright respond that vaccines were unsafe and should be avoided, even though a significant portion of Americans hold that belief [5]. Nevertheless, the neutral responses provided by VAs did not explicitly highlight the safety of vaccines and may leave room for doubt if a user is already skeptical of vaccination.

Compared to the direct responses, the websites provided by VAs were much less likely to be outright supportive of vaccination, although importantly, no website had an explicitly negative connotation. Still, many of the websites with neutral connotations left room for doubt, with titles that highlighted adverse events (eg, “How long should COVID vaccine side effects last?”), implied a degree of social stigma around vaccination (eg, “Should you tell people you got the COVID-19 vaccine? Here’s what to consider”), or focused on contraindications to vaccination (eg, “health questions to consider before taking the COVID-19 vaccine”).

As society progresses further into the digital age, the way health information is presented to and consumed by the public is changing. Over the past decade, we have seen how health information is increasingly disseminated through the internet and social media, with significant implications for public safety. This has been especially relevant during the COVID-19 pandemic, where efforts to educate the public around safety measures, including masking and vaccination, have been fraught by the spread of misinformation and disinformation online.

Previous studies have highlighted pitfalls associated with the use of VAs as sources of health information, which often offer counsel that is inaccurate or incongruous with official recommendations [6-14]. Our study adds to this body of work by examining the responses of VAs to questions about the COVID-19 vaccine. This work is novel in that it explores the responses of VAs to information-seeking questions that can be answered by medical evidence (eg, “Is the COVID vaccine safe?”) and it also asks them to respond to recommendation-seeking questions (eg, “Should I get the COVID vaccine?”).

### Limitations

Our study is limited by the exclusion of many survey responses due to manual entry and incomplete or duplicate responses, suggesting potential for improvements in data collection in the future. Unfortunately, this is a risk inherent in survey-based research conducted on the internet (eg, increasing prevalence of survey bots). To mitigate this risk, substantial effort was undertaken to clean and ensure the validity and trustworthiness of the data that was included in the final analysis. Additional limitations include the overrepresentation of Apple devices using Siri, which may skew the types of responses we received, and the large percentage of respondents who were from coastal regions, highly educated and supportive of COVID-19 vaccination. It is possible that VAs could tailor their responses based on their users’ demographics and search history, and future work should aim to address this question by including respondents of diverse backgrounds. Lastly, our finding that VAs were more likely to respond directly to an information-seeking query than to a recommendation-seeking query was based on 2 COVID-19–related questions. Future work should investigate whether this behavior of VAs is generalizable to other health-related questions.

### Conclusions

Despite being an increasingly popular way for the public to access health information, current state VAs could be a source of ambiguous or potentially biased information. Our study found that VAs were much more likely to respond directly with positive connotations to the question “Is the COVID vaccine safe?” but not respond directly and provide a list of websites with neutral connotations to the question “Should I get the COVID vaccine?” These findings add to our growing understanding of both the opportunities and pitfalls of VAs in supporting public health information dissemination, warranting further evaluation by public health professionals, technologists, and policymakers.

## Abbreviations

VA: voice assistant

## Data Availability

The data sets generated and analyzed during this study are available from the corresponding author on reasonable request.
